# MATCH‐IT: A decision support tool for multiple comparisons presenting data from network meta‐analysis to facilitate guideline development

**DOI:** 10.1002/gin2.70003

**Published:** 2024-09-17

**Authors:** Birk S. Hunskaar, Per O. Løvsletten, Frankie Achille, Anja F. Heen, Thomas Agoritsas, Per O. Vandvik

**Affiliations:** ^1^ Institute of Health and Society, Faculty of Medicine University of Oslo Oslo Norway; ^2^ Department of Medicine Lovisenberg Diaconal Hospital Oslo Norway; ^3^ MAGIC Evidence Ecosystem Foundation Oslo Norway; ^4^ Department of Medicine, Division of General Internal Medicine University Hospitals of Geneva Geneva Switzerland; ^5^ Department of Health Research Methods, Evidence and Impact McMaster University Hamilton Ontario Canada

**Keywords:** guideline development, multiple comparisons, network meta‐analysis, NMA

## Abstract

**Introduction:**

Network meta‐analysis (NMA) provides unprecedented opportunities to compare multiple treatment options (multiple comparisons) and are increasingly being used to inform clinical practice guidelines. However, the overwhelming amount of data generated from NMAs is challenging to present in comprehensible overviews for end‐users, guideline panelists included. Acknowledging these challenges, we developed MATCH‐IT—an interactive evidence summary displaying NMA results for multiple comparisons. In this study, we conducted user‐testing and further developed MATCH‐IT to support guideline panels in moving from NMA evidence to recommendations.

**Methods:**

We user‐tested the tool with guideline panelists and observed the use of the tool in panel meetings. User‐testing sessions and guideline meetings were recorded, transcribed, and analyzed. The analysis informed the iterative development in the tool.

**Results:**

We included four guideline panels and tested the tool with 15 panelists (four chairs, four methodologists, five clinical experts and two patient partners). User testing revealed both positive aspects and limitations of the tool. Interactivity allowed for dynamic display of the evidence during panels meetings and was highlighted as valuable. Further, participants felt that the tool provided overview of complex evidence, further facilitated by categorization of effects through colour coding. The inclusion of information on burden of treatment was highlighted as relevant and valuable. Regarding limitations, some users had issues discovering the interactive features. Earlier versions of MATCH‐IT did not include sufficiently detailed information, such as the imprecision of effect estimates, which the users felt was needed for decision making. These findings led to refinements of the tool, including a new tutorial, inclusion of confidence intervals, and a new layer displaying more detailed information.

**Discussion:**

Our study suggests that MATCH‐IT may play a role in facilitating guideline development by easing understanding of NMA evidence and alleviating information overload in guideline panelists.

## Key points

*What is already known on this topic?*

- NMAs have become an increasingly important data source in development of clinical practice guidelines.
- NMAs produce large amounts of data, presenting challenges to both presentation and interpretation of NMA evidence.
- In the context of guideline panel meetings, time for review and interpretation of evidence is limited.

*What this study adds?*

- We developed MATCH‐IT, an interactive GRADE evidence summary displaying NMA data for multiple comparisons through user‐testing and deployment in guideline panel meetings.
- MATCH‐IT underwent several rounds of updates to accommodate the needs of guideline panelists for evidence review individually and in the context of guideline panel meetings.
- MATCH‐IT may be used to present and review NMA evidence in clinical guideline development, by easing understanding of NMA evidence and alleviating information overload in guideline panelists.
- MATCH‐IT can be viewed and interacted with using this link.

## 1 INTRODUCTION

The scope of modern medicine and the increasing volume of evidence produced requires that clinical practice guidelines make recommendations among a myriad of viable treatment options. Network meta‐analysis (NMA) lends itself nicely to this task, combining both direct and indirect evidence to compare multiple treatment options (multiple comparisons).^1^ As a result, systematic reviews with NMA have surged in popularity and are increasingly being used as the basis for guideline development.^2^, ^3^, ^4^ However, the extensive data output from NMA is difficult to present in comprehensible formats and little guidance exits for researchers on how to best present results in accessible formats for relevant end users—guideline panelists included.^5^

For pairwise comparisons, progress has been made by structuring data into evidence summaries—for example, in the form of Grading of Recommendations, Assessment, Development and Evaluation (GRADE) Summary of Findings (SoF) tables.^6^ We believe NMA and multiple comparisons should be held to the same standards, ideally by presenting evidence in *structured evidence summaries* displaying both benefits *and* harms of the alternative treatment options, presented in absolute effect estimates, as well as certainty of the evidence.^7^ Very few current systematic reviews with NMA include such evidence summaries, often reporting only one outcome at a time^8^, ^9^ or leaving out certainty ratings.^10^, ^11^, ^12^ Thus, readers must manoeuvre between numerous formats to gather the information necessary for issuing recommendations. In the face of large NMAs, this exercise may induce information overload, and is particularly impractical in guideline panel meetings with limited time for presentation and discussion of the evidence.

In response to these challenges, the MAGIC Evidence Ecosystem Foundation (https://www.magicevidence.org) has developed a new digital and interactive decision support tool displaying structured evidence summaries for multiple comparisons from NMAs—henceforth called MATCH‐IT.^13^ Over the last few years, MATCH‐IT has been tested and developed through user testing studies on relevant target populations.^14^ In this study, we aimed to evaluate and improve MATCH‐IT specifically in the context of guideline development; both in how it is received by guideline panelists and how it performs in guideline panel meetings when moving from NMA evidence to recommendations.

## 2 METHODS AND MATERIALS

### 2.1 Overview

**Figure 1:**
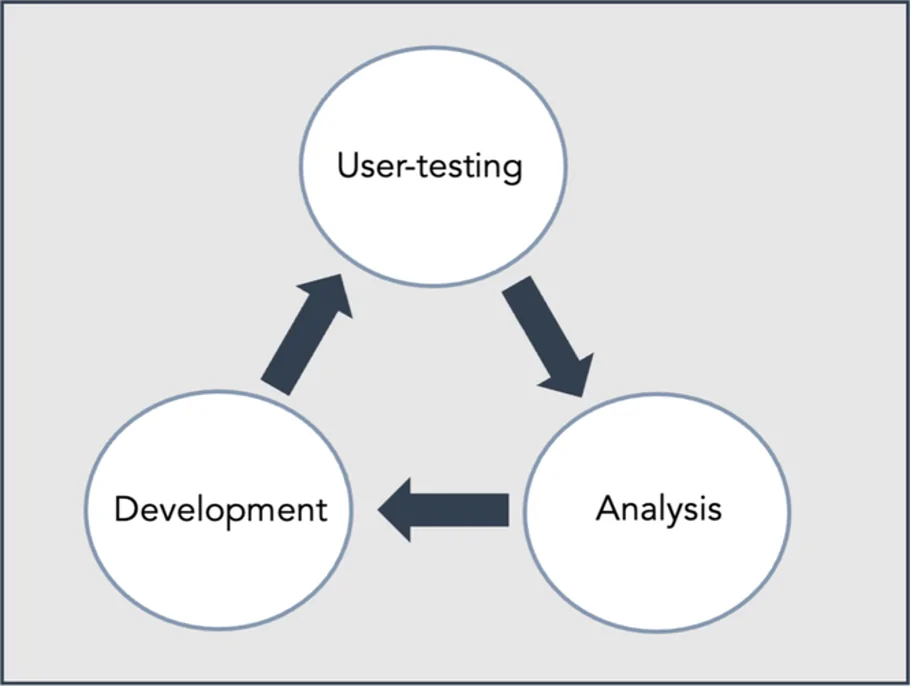
Illustration of an iterative cycle.

The study took place from February 2021 to January 2023 and was conducted by researchers from MAGIC and the University of Oslo. The study was deemed exempt from formal approval by a regional ethics committee. For reporting, we adhered to the Standards for Reporting Qualitative Research (SRQR).^15^ Drawing inspiration from earlier research and innovation projects,^16^, ^17^ we applied principles of usability testing and user‐centered design^18^, ^19^ in an iterative development process to evaluate and develop the tool (Figure 1). One iterative cycle consisted of the following steps:

1. *User‐testing*: included both (a) user‐testing sessions with guideline panelists and (b) observations of the use of the tool in guideline panel meetings.
2. *Data analysis*: transcription of user‐testing sessions and guideline panel meetings before data analysis.
3. *Development*: refinements were made based on our analysis.

### 2.2 MATCH‐IT features at inception of study

MATCH‐IT presents data in accordance with GRADE methods and is an interactive SoF (iSoF) table.^20^ It is a multi‐layered tool, allowing users to manoeuvre between layers to explore evidence at varying degrees of detail. Entering the tool, the user is asked to select baseline risk if a risk stratification of the patient population has been performed. If not, the user can select outcomes to build the iSoF table. Moving further, users can click on specific pairwise comparisons to enter a display showing a graphical visualization of the effects. MATCH‐IT also includes interactive features allowing the user to shuffle, add, and remove interventions and outcomes to tailor the display to their needs. Figure 3 (under results) shows a screenshot of one of the tested versions of the tool, while an interactive version of the latest tool can be accessed through a recent NMA publication.^21^

### 2.3 Setting, guideline processes and recruitment of participants

We included national or international guideline panels using GRADE methods and a systematic review with NMA as a basis for their guideline, aiming both to observe the use of the tool during panel meetings and conduct user‐testing sessions with guideline panelists. Guideline panels were selected based on (a) if the chairs were willing to use MATCH‐IT in the guideline process, after an initial presentation of its features, and (b) whether the NMA underpinning the guideline was sufficiently complex to challenge MATCH‐IT in accommodating the data set—ideally with multiple interventions and outcomes.

For user‐testing sessions, we recruited a convenience sample of participants from the guideline processes. Guideline panels consist of individuals with different expertise and roles in the panel (e.g., methodologists, clinicians, patient partners); we aimed to capture this diversity in our sample, as well as have a balance in gender representation.

Both user‐testing sessions and guideline panel meetings took place digitally and were videorecorded.

### 2.4 User‐testing sessions

We conducted user‐testing sessions in preparation for or as a follow‐up after the guideline meetings. We started gathering background information and explaining the purpose of the session, before asking participants to freely explore the tool and its content. We asked participants to think aloud, sharing their instant experiences and thoughts while interacting with the tool. Finally, we performed a semi‐structured interview to explore their experience and elicit feedback on specific functions. We used a standard interview guide, piloted before initiation of the study (File S1), although the focus and structure of the sessions was pragmatically changed to fit each participant.

All user testing sessions were transcribed performed manually or assisted by a GDPR‐approved AI transcription software (https://www.amberscript.com/en). In the latter cases, we quality‐checked all transcripts.

### 2.5 Guideline panel meetings

In preparation for the guideline meetings, panel chairs were offered a walkthrough on how to use the tool. The panel chairs had the freedom to use the tool the way best saw fit and were solely responsible for how and to what extent they would include MATCH‐IT in the guideline meetings. We did not actively participate in the meetings. Afterwards, we condensed the parts of the meetings where MATCH‐IT was used into a narrative summary.

### 2.6 Data analysis

All transcripts were anonymized and uploaded to an online qualitative research software—*Atlasti* (https://atlasti.com/). We applied principles of directed content analysis,^22^, ^23^ and searched the transcripts for*units of meaning*—phrases or interactions describing user experiences—which we coded along two deductively derived frameworks: a modified version of Morville's Honeycomb for user experience,^24^ and a framework for capturing the quality of the experience^16^ (File S2). Additionally, we added inductive codes to each meaning unit to describe the user experiences more precisely, as well as geographical tags to pinpoint where in the tool the users had the experiences.

After the initial coding process, we transferred the material into Microsoft Excel to hierarchically organize the codes into a coding tree, searching for commonalties across user experiences and synthesizing inductively derived codes into larger categories and linking them to the deductive frameworks (File S3). The categories were checked against their original phrases to ensure their validity. All steps in data analysis were conducted and reviewed by two researchers. In the case of disagreements, these were discussed, and a consensus was reached.

### 2.7 Development

Based on the data analysis, numerous suggestions for updates were prepared and discussed in research team meetings. The research team discussed the results and jointly decided on which changes to prioritize in the next iteration.

## 3 RESULTS

### 3.1 Guideline processes and participants

We included four guideline processes,^25^, ^26^, ^27^, ^28^ two of which were informed by the same NMA.^29^ We observed the use of MATCH‐IT in three guideline meetings and recruited 15 participants to user‐testing (Table 1).

**Table 1:** Guideline and participant characteristics.

Guideline characteristics			
Guideline topic (link to MATCH‐IT)	No of outcomes	No of interventions	No of panelists interviewed (%)
Lipid‐lowering drugs (V1)		4	6 (40%)
Induction therapy after heart transplant (V2.0, V2.1)	13	3	4 (26%)
DMARDs after failed TNF‐α‐I therapy Australia (V3)	6	15	3 (20%)
DMARDs after failed TNF‐α‐I therapy Canada (V4)	6	15	2 (13,3%)

Participant characteristics	
Characteristic	Number (%)
Role/perspective	
Panel chair/co‐chairs	4 (27%)
Clinical experts/content experts	5 (33%)
Guideline methodologists, systematic review/NMA experts	4 (27%)
Patient partners	2 (13%)
Gender representation	
Women	9 (60%)
Men	6 (40%)
Age	
Median (range)	55 year (29–70)

### 3.2 MATCH‐IT versions

We performed three rounds of iterative development, resulting in four versions of MATCH‐IT. We made several updates over the course of the study; both usability fixes aiming to address programming bugs and errors as well as more conceptual changes such as adding new data elements. Table 1 provides hyperlinks to all MATCH‐IT tools that were developed and used, while File S2 provides a schematic and visual overview of the changes that were made. Below, we present a summary of main findings and changes made across the development process.

### 3.3 Results from content analysis

**Figure 2:**
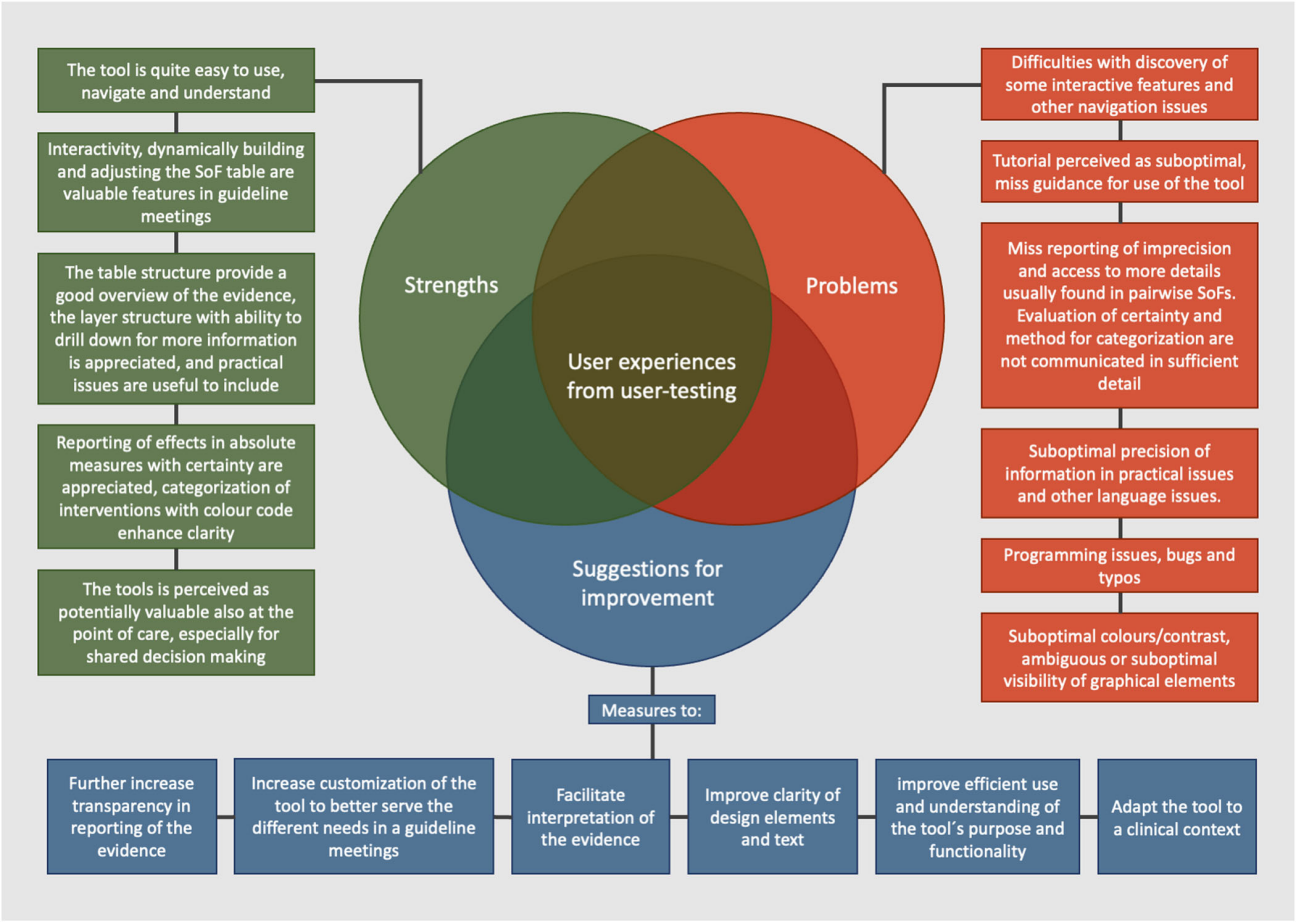
Overview of user experiences.

We coded and analyzed 553 meaning units in total, of these 472 (85%) were distributed within three out of eight Morville categories (usefulness, understandability and usability). To build our coding tree, we used strengths, problems, and suggestions for improvement—all derived and modified from the quality of experience model^16^—as overarching themes (File S3). Figure 2 shows a visual overview of the user‐experiences.

### 3.4 Strengths

We identified aspects of the tool that functioned well and/or received positive feedback across iterations. First, building the iSoF table by selecting outcomes was intuitive to users. This feature, together with other interactive features, allowed for the sequential review of the evidence one outcome at a time, while at the same time building and displaying an overview. This was reported and observed to be useful in guideline meetings (Panel meetings round 3 and 4).

“I think it was just easy. Like, you don't need a tutorial on how to use the tool […] it was very intuitive.”

(Panel cochair)

“It was useful, putting it together and shifting them around, […] so that you were only looking at the relevant questions.”

(Clinical expert)

Participants commented that MATCH‐IT provided a clear overview of the evidence, without inducing information overload by cluttering the table with details. Several participants reported that the table had the potential to replace the otherwise common practice of sequentially reviewing multiple SoF tables for pairwise comparisons in guideline panel settings.

“It's always a challenge to funnel a lot of information into a palatable summary, I think the tool does that very nicely.”

(Methods expert)

“It's a method of presenting […] complex comparisons in a way that actually manageable, because the other ways of presenting NMA data which is with these two‐by‐two tables […] they just can't understand what I'm saying.”

(Methods chair)

“I think this would have saved the [panel chair] a lot of work explaining these slides.”

(Clinical expert)

Overall, participants revealed that they thought the tool could facilitate guideline panel discussions. This was supported by observations in real‐life guideline meetings, particularly when chairs were well‐prepared and had developed an effective choreography for the use of MATCH‐IT, tailoring the tool to fit the ongoing discussion of the panel (Panel meeting round 3).

Regarding the practical issues module, participants commented that it highlighted and addressed relevant considerations that are not necessarily part of a typical guideline process. A common opinion was that this module may trigger discussions in the panel about additional aspects to consider when deciding on recommendations.

“It's actually one of the best things about this. […] This part brings up topics that either you might forget about, or it might be difficult to address.”

(Patient partner)

“Because it addresses information that's not addressed by the guidelines […], Cost wasn't addressed, storage wasn't addressed. And how is it how is it administered? Wasn't addressed there. So, it would be it's nice to have a quick reference to these things for this guideline.”

(Clinical expert)

### 3.5 Limitations

We also identified several limitations with the tool, some of which concerned general user‐tool interaction. While building the iSoF table was intuitive, the interactive features—allowing for customization of the table – were often overlooked. Further, the tutorial available in the tool was reported to be difficult to understand and going too fast.

“[Removing interventions] is a good function. But that just doesn't feel like something you could do.”

(Methodologist)

“It's not clear. Honestly, it didn't help me using the table. [commenting on tutorial]”

(Clinical expert)

More specifically for guideline processes, participants often expressed that they needed access to more information. Although GRADE certainty of evidence ratings taking imprecision into account were displayed, participants expected and wanted confidence intervals to be reported, as well as relative effect estimates and number of studies and participants underpinning the comparisons. In one guideline panel meeting (*round 2*), panelists and chairs felt uncomfortable using MATCH‐IT or publishing it along with the guideline, as they felt that that version of MATCH‐IT—without confidence intervals—failed to convey the true uncertainty and imprecision in the data.

“I think it's essential for a guideline group to have those confidence intervals.”

(Clinical expert)

“I want to find the information about the number of people included in the trails […] or the number of trails….”

(Methodologist)

“[…] It's a little bit bizarre to bold the point estimate when you have so much uncertainty around the confidence interval, […] In our case, in fact, the confidence interval was more important than the point estimates.”

(Methods chair)

### 3.6 Key developments

We made several updates to the tool in response to these challenges. An early development was to update the tutorial, going from a continuous video to a stepwise approach with explanatory text providing information on both the content and functionality of MATCH‐IT.

Due to the information needs of the panel, we made several major additions to the tool. Confidence intervals were added to the effect estimates displayed in the table, and the tool was furnished with a new 3rd layer, now including relative effect estimates and the number of studies and participants underpinning the pairwise comparison.

**Figure 3:**
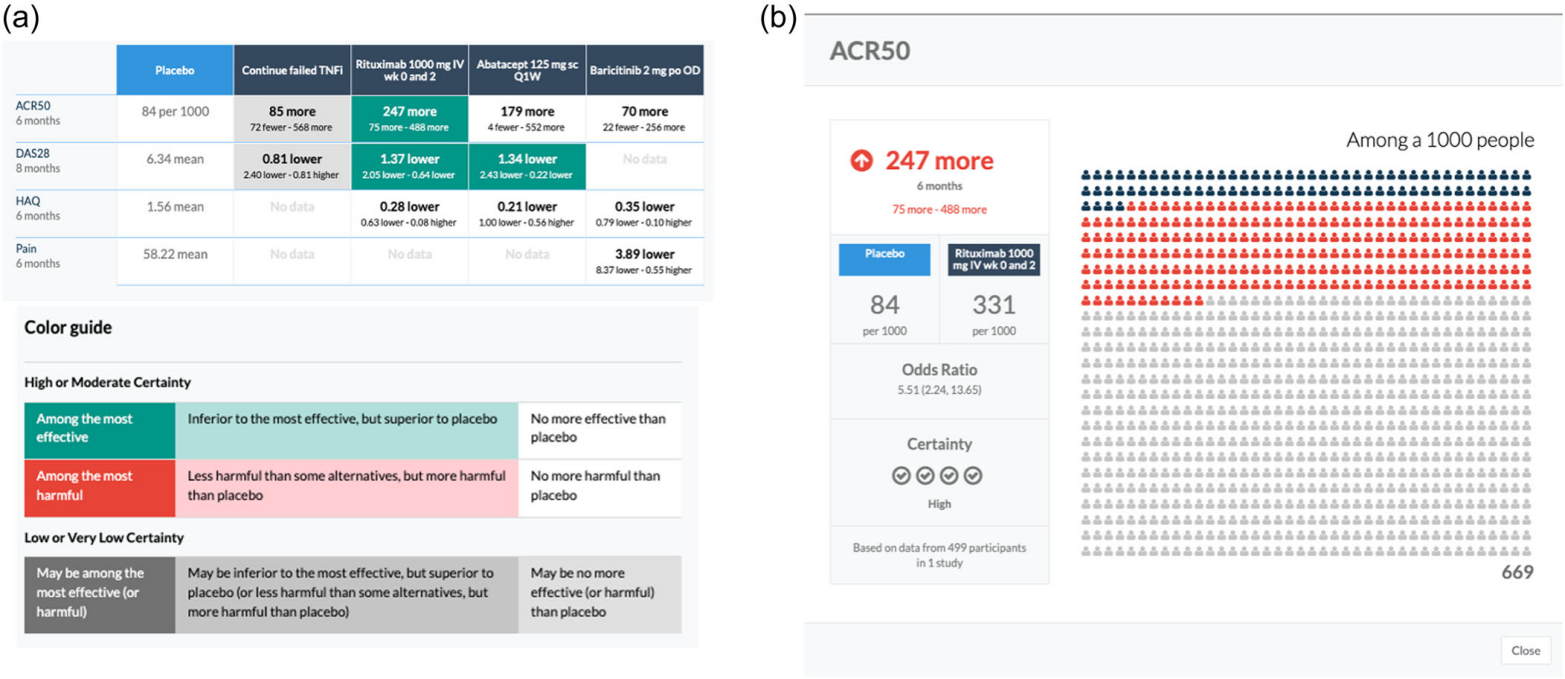
(a) Crop of the main table and colour guide. (b) Updated 3rd layer.

To better visually differentiate between interventions, categorization of interventions through colour coding was added. The major driver behind this change was observations in a parallel user‐testing study ran on clinicians.^30^ The colour coding was based on the GRADE minimally contextualized approach^31^ and the gradient colour variant developed by Phillips et al.^32^ Figure 3 shows these changes in MATCH‐IT presenting data from the two latest guidelines.

### 3.7 Evaluation of updates in the tool

The inclusion of confidence intervals did not elicit explicit positive feedback from participants, although no further requests to display them were made. Further, they were seen to fit into the workflow of panel meetings (rounds 3 and 4), by being referenced in the panel discussions on several occasions*.* The updated 3rd layer was positively received, although not widely used; participants were seemingly satisfied that the information was available but did not check this for every comparison. In one panel meeting, participants asked the panel chair to display the 3rd layer for comparisons they were interested in on several occasions (round 3).

“It [3rd layer] just gives me a clearer picture of the numbers.”

(Clinical expert)

As for the colour coding, feedback from user‐testing sessions was mixed. Some participants reported the rationale behind the categorization to be difficult to grasp, while others highlighted the colour coding as a key feature of the tool. In the panel discussions, the colour coding was not seen to cause any friction or problems after a brief explanation by panel chair(s) (rounds 3 and 4).

“I think this [color guide] is hard for people to understand. […] there's so much theory behind this.”

(Method's co‐chair)

“If you look at the colors […] and start putting things in intuitive order, you start seeing color patterns. […] this made intuitive sense to everyone [guideline panelists]”

(Methods chair)

The updated tutorial was shown to provide relevant instruction to individual participants, although participants generally preferred to start interacting with the tool without first accessing the tutorial.

“This is great though, there's a lot of immediate questions that jump out when you don't go through the tutorial.”

(Methods chair)

## 4 DISCUSSION

### 4.1 Main findings

Our study shows the technical and conceptual development of a decision support tool for multiple comparisons to fit the needs of guideline panelists and facilitate guideline development. We uncovered both strengths and limitations of the tool leading to several refinements, with some unresolved issues such as communicating the tool functions to the user. Overall, our analysis suggests that the most notable features of MATCH‐IT in the context of guideline development are (1) its ability to provide an overview of the evidence, further facilitated by categorization with colour codes, (2) the interactive features allowing for dynamic adjustment of the display of the evidence and (3) the opportunities to view information on burden of treatment.

### 4.2 Strengths and limitations

MATCH‐IT was user‐tested on panel members and was employed in real‐life guideline panel meetings. Further, we employed methodological measures to increase the validity of our analysis, as all transcripts were coded and reviewed in duplicate. Our triangulation of data sources—that is, using both interviews and guideline panel meetings—, also contributed to the rigour of the study.^33^ The NMAs underpinning the guidelines included in the study varied in key aspects such as number of interventions, outcomes, and certainty ratings, challenging MATCH‐IT to accommodate a variety of datasets. The latest versions of MATCH‐IT—where we observed the most successful employments of the tool—presented data from a large network with 15 interventions across 6 outcomes.

The study also has some limitations. First, we recruited participants for user‐testing through a convenience sampling approach and had several nonresponders. Although our sample was balanced with regard to representation of roles and gender, this may have biased our sample towards the most willing and enthusiastic panelists. Secondly, representing a major drawback of the study, all guideline panels had the support of the research team in planning the panel meetings. Thus, we cannot attest to how MATCH‐IT works in stand‐alone guideline panels. Finally, we were unable to successfully employ MATCH‐IT in the first two panel meetings in our study. For one meeting this was due to time constraints, while for another, this was due to chairs and panelists finding MATCH‐IT unfit at the time. Our experience with using MATCH‐IT during panel meetings therefore remains limited. We made several updates to the tool following these experiences, and MATCH‐IT was used with considerably more success in the following meetings.

### 4.3 Implications for research and practice

In addition to this study, MATCH‐IT has been evaluated through studies on other target populations. Most notably, we have ran a user testing study employing the same methodology as in this work on another key target group: physicians, finding that MATCH‐IT showed promise in communicating NMA results to this group.^30^ With some differences, these findings were echoed for guideline panelists in this study. Panelists typically have higher research literacy, requiring more detailed information to appropriately make recommendations. This is exemplified by our inclusion of confidence intervals and a more detailed 3rd layer in the present study. We have also performed a web‐based survey of 3rd year medical students, exposing them to different versions of MATCH‐IT.^14^ Beyond finding that the medical students could use and understand MATCH‐IT to interpret NMA evidence, the study serves as an example of how quantitative studies can be used to evaluate the comparative merit of different design alternatives or presentation formats.

The variable success in using MATCH‐IT in panel meetings illustrates that several challenges remain in this context. Despite our advances with MATCH‐IT and our measures to alleviate information overload, we recognize that guideline development based on NMA evidence remains challenging. One example is a recent guideline for temporomandibular joint disorders,^34^ where the NMA included 58 interventions.^35^ For this guideline, MATCH‐IT was not used, both because we felt uncertain that the tool could accommodate a data set of its size, but also because the number of recommendations required refinements to current GRADE approach. Furthermore, development of several features remains for MATCH‐IT to function optimally in the evidence ecosystem. First, while there was recently developed a solution for automated data import—where NMA results structured in R can be ported into to the back‐end data structure of MATCH‐IT—this is not possible without support from the research team. Second, NMA researchers may want to use other tools for evidence synthesis^36^, ^37^ before using MATCH‐IT for presentation purposes, and no solution for external integration with such tools exists. Thirdly, MATCH‐IT will have to accommodate both current and future developments within the field of NMA. One example is NMA with multicomponent interventions—CNMA^38^, ^39^—which we currently have no strategy for presenting within MATCH‐IT. MATCH‐IT is currently undergoing integration into MAGICapp, and several features may be developed during this process, while other features may need to be developed down the line.

Concerning more general implications for practice, the tool has already been published as part of systematic reviews and guidelines—including those developed in the present study—demonstrating how it can be practically applied.^25^, ^40^, ^41^, ^42^, ^43^ Most notably, MATCH‐IT was published in WHO living guidelines for COVID‐19 therapeutics.^43^ Here, incoming studies informed the ongoing NMA^44^ which fed further into the rapidly updated CPG demonstrating a breakthrough for “living evidence”.^45^

## 5 CONCLUSION

Our study suggests that MATCH‐IT may play a role in facilitating guideline development by easing understanding of NMA evidence as well as alleviating information overload in guideline panelists. More development is needed for MATCH‐IT to function optimally in guideline development.

## AUTHOR CONTRIBUTIONS

All authors made substantial contributions to the study. Birk S. Hunskaar and Per O. Løvsletten draughted the interview guide, performed the interviews and the analysis and sketched suggestions for updates. Frankie Achille designed the MATCH‐IT prototype and implemented all updates to the tool. Anja F. Heen and Thomas Agoritsas contributed to the first prototype of MATCH‐IT and provided guidance on the interview guide and qualitative research methods as well as feedback on the manuscript. Birk S. Hunskaar and Per O. Løvsletten draughted the manuscript, while Frankie Achille, Anja F. Heen, Thomas Agoritsas and Per O. Vandvik provided feedback and reviewed. Per O. Vandvik is project lead and guarantor of this article. All listed authors meet authorship criteria and no others meeting them have been omitted.

## CONFLICT OF INTEREST STATEMENT

Birk S. Hunskaar and Per O. Løvsletten perform research on the MATCH‐IT tool, within the MAGIC Evidence Ecosystem Foundation (MAGIC/www.magicevidence.org). Per O. Vandvik and Thomas Agoritsas are respectively CEO and Chair of the Board of MAGIC Evidence Ecosystem Foundation and may as such have intellectual conflicts of interests related to this research.

## ETHICS STATEMENT

The MATCH‐IT project was deemed exempt from a regional ethics committee. All participants signed consent forms before participating in interviews. All recordings of interviews and meetings were stored on a secure storage device before being immediately transferred to a secure server.

## Supporting information

Supporting information.

Supporting information.

Supporting information.

## Data Availability

The data that support the findings of this study are available on request from the corresponding author. The data are not publicly available due to privacy or ethical restrictions.
