# Supplementary material for: MATCH‐IT: A decision support tool for multiple comparisons presenting data from network meta‐analysis to facilitate guideline development
Source: Clin Public Health Guidel. 2024 Sep 17;1(4):e70003. doi: 10.1002/gin2.70003 (PMC13563384; doi:10.1002/gin2.70003)
Supplement: Supplementary file 1 [file GIN2-1-e70003-s001.pdf]

Risk selection screen / 1st layer

Choose baseline risk

Already receiving statins without another lipid-lowering drug

Adults with low cardiovascular (CV) risk factors

Adults with moderate cardiovascular (CV) risk factors

Adults with high cardiovascular (CV) risk factors

Statins intolerant population without lipid-lowering drugs

Adults with low cardiovascular (CV) risk factors

Adults with moderate cardiovascular (CV) risk factors

Adults with high cardiovascular (CV) risk factors

MATCH-IT is an interactive tool designed to help you explore the evidence

Access MATCH-IT

How do I use MATCH-IT

Developed by MAGIC Evidence Ecosystem Foundation

Tutorial

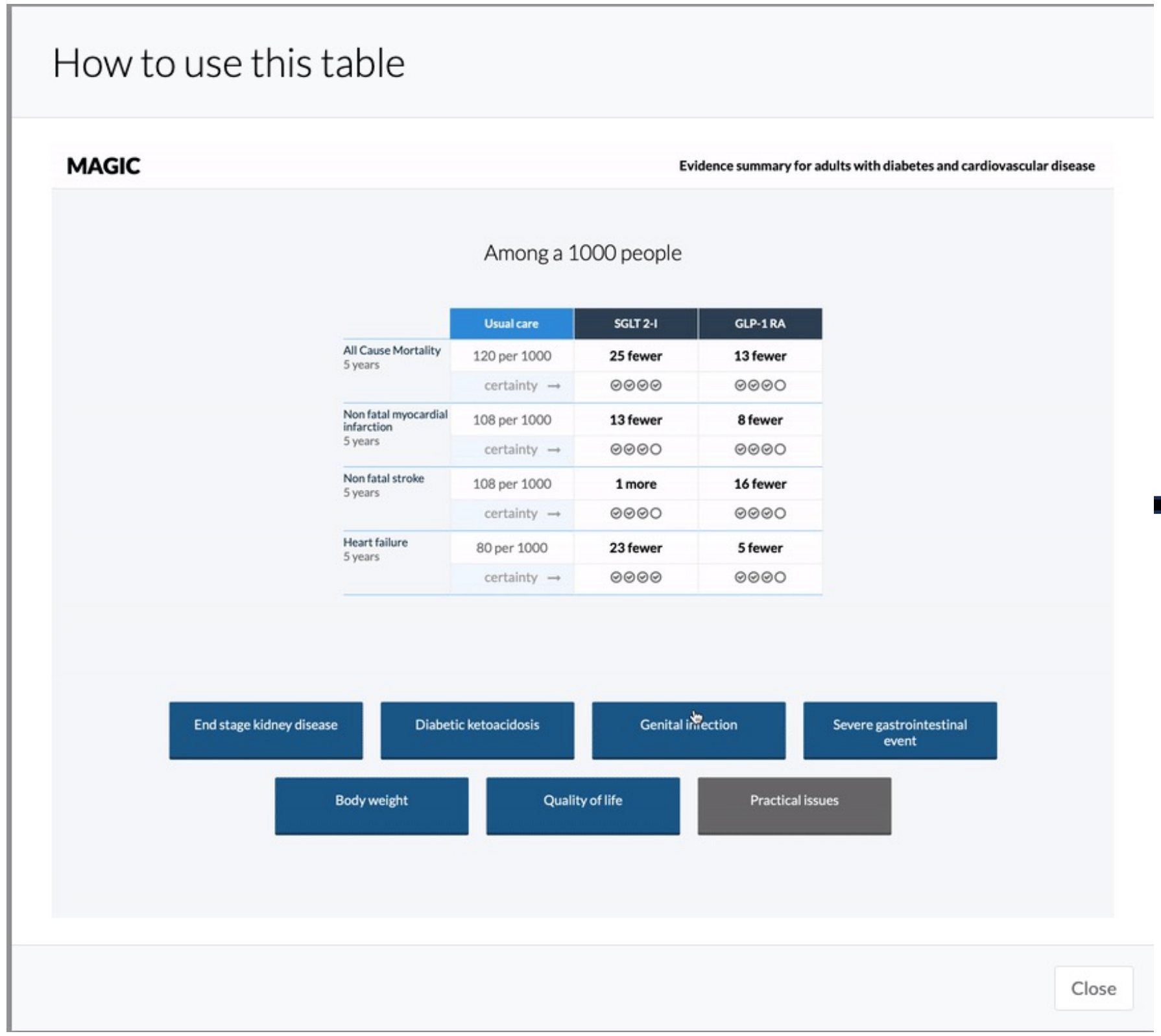
How to use this table
MAGIC
Evidence summary for adults with diabetes and cardiovascular disease
Among a 1000 people
Usual care
SGLT 2-I
GLP-1 RA
All Cause Mortality
5 years
120 per 1000
25 fewer
13 fewer
certainty
Non fatal myocardial infarction
5 years
108 per 1000
13 fewer
8 fewer
certainty
Non fatal stroke
5 years
108 per 1000
1 more
16 fewer
certainty
Heart failure
5 years
80 per 1000
23 fewer
5 fewer
certainty
End stage kidney disease
Diabetic ketoacidosis
Genital infection
Severe gastrointestinal event
Body weight
Quality of life
Practical issues
Close

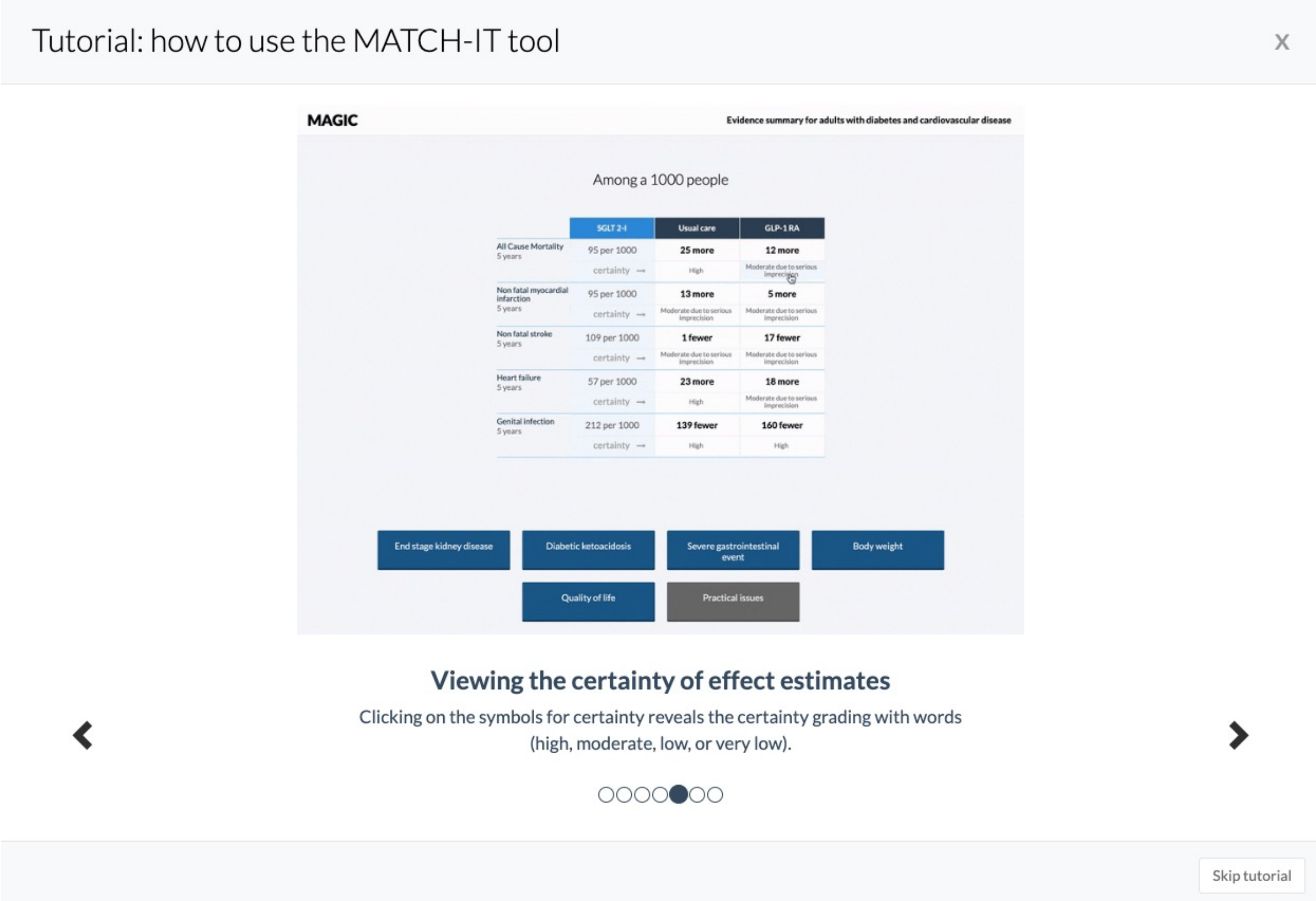
Tutorial: how to use the MATCH-IT tool
x
MAGIC
Evidence summary for adults with diabetes and cardiovascular disease
Among a 1000 people
End stage kidney disease
Diabetic ketoacidosis
Severe gastrointestinal event
Body weight
Quality of life
Practical issues
Viewing the certainty of effect estimates
Clicking on the symbols for certainty reveals the certainty grading with words (high, moderate, low, or very low).
Skip tutorial

2nd layer / main table

Among a 1000 people

	No lipid-lowering drug	PCSK9	Ezetimibe	PCSK9 + Ezetimibe
All-cause mortality<br>5 years	23 per 1000	No important difference	No important difference	No important difference
	certainty →	⨁⨁⨁⨁	⨁⨁⨁⨁	N/A
Cardiovascular mortality<br>5 years	15 per 1000	No important difference	No important difference	No important difference
	certainty →	⨁⨁⨁⨁	⨁⨁⨁⨁	N/A
Non-fatal MI<br>5 years	76 per 1000	**15 fewer**	**11 fewer**	**24 fewer**
	certainty →	⨁⨁⨁◯	⨁⨁⨁◯	N/A
Non-fatal stroke<br>5 years	59 per 1000	**14 fewer**	**7 fewer**	**19 fewer**
	certainty →	⨁⨁⨁◯	⨁⨁◯◯	N/A

How to use this table

Among a 1000 people

	No IT	Thymoglobulin	Basiliximab
Mortality<br>1-Year (RCT)	59 per 1000	**85 more**<br>28 fewer - 411 more	**59 more**<br>35 fewer - 357 more
Mortality<br>1-Year (Obs)	109 per 1000	**3 fewer**<br>11 fewer - 5 more	**11 more**<br>2 more - 21 more
Mortality<br>5-Year (Obs)	182 per 1000	**19 more**<br>51 fewer - 113 more	**23 more**<br>68 fewer - 157 more
Rejection<br>6-Month (RCT)	290 per 1000	**126 fewer**<br>256 fewer - 192 more	**69 more**<br>135 fewer - 344 more

2nd layer / main table – color coding

Among a 1000 people

	No IT	Thymoglobulin	Basiliximab
Mortality (RCT 1yr)<br>1-Year	59 per 1000	**85 more**<br>28 fewer - 411 more<br>Very low certainty	**59 more**<br>35 fewer - 357 more<br>Very low certainty
Mortality (Obs 1yr)<br>1-Year	109 per 1000	**3 fewer**<br>11 fewer - 5 more<br>Very low certainty	**11 more**<br>2 more - 21 more<br>Low certainty
Mortality (Obs 5yr)<br>5-Year	182 per 1000	**19 more**<br>51 fewer - 113 more<br>Very low certainty	**23 more**<br>68 fewer - 157 more<br>Very low certainty
Rejection (RCT 6mo)<br>6-Month	290 per 1000	**126 fewer**<br>256 fewer - 192 more<br>Very low certainty	**69 more**<br>135 fewer - 344 more<br>Very low certainty

Color code guide

High or Moderate Certainty (GRADE)

Among the most effective	Inferior to the most effective, but superior to No IT	No more effective than No IT
Among the most harmful	Less harmful than some alternatives, but more harmful than No IT	No more harmful than No IT

Low or Very Low Certainty (GRADE)

May be among the most effective	May be inferior to the most effective, but superior to No IT	May be no more effective than No IT
May be among the most harmful	May be less harmful than some alternatives, but more harmful than No IT	May be no more harmful than No IT

	Placebo	Abatacept 125 mg sc Q1W	Switch in TNFi route SC->IV	Rituximab 1000 mg IV wk 0 and 2	Baricitinib 2 mg po OD
ACR50<br>6 months	84 per 1000	**179 more**<br>4 fewer - 552 more	**21 more**<br>78 fewer - 518 more	**247 more**<br>75 more - 488 more	**70 more**<br>22 fewer - 256 more
DAS28<br>8 months	6.34 mean	**1.34 lower**<br>2.43 lower - 0.22 lower	**1.01 lower**<br>2.99 lower - 0.99 higher	**1.37 lower**<br>2.05 lower - 0.64 lower	No data
HAQ<br>6 months	1.56 mean	**0.21 lower**<br>1.00 lower - 0.56 higher	No data	**0.28 lower**<br>0.63 lower - 0.08 higher	**0.35 lower**<br>0.79 lower - 0.10 higher
Pain<br>6 months	58.22 mean	No data	No data	No data	**3.89 lower**<br>8.37 lower - 0.55 higher

Color guide

High or Moderate Certainty

Among the most effective	Inferior to the most effective, but superior to placebo	No more effective than placebo
Among the most harmful	Less harmful than some alternatives, but more harmful than placebo	No more harmful than placebo

Low or Very Low Certainty

May be among the most effective (or harmful)	May be inferior to the most effective, but superior to placebo (or less harmful than some alternatives, but more harmful than placebo)	May be no more effective (or harmful) than placebo

2nd layer / main table – color coding

	Placebo	Abatacept 125 mg sc Q1W	Switch in TNFi route SC->IV	Rituximab 1000 mg IV wk 0 and 2	Baricitinib 2 mg po OD
ACR50<br>6 months	84 per 1000	**179 more**<br>4 fewer - 552 more	**21 more**<br>78 fewer - 518 more	**247 more**<br>75 more - 488 more	**70 more**<br>22 fewer - 256 more
DAS28<br>8 months	6.34 mean	**1.34 lower**<br>2.43 lower - 0.22 lower	**1.01 lower**<br>2.99 lower - 0.99 higher	**1.37 lower**<br>2.05 lower - 0.64 lower	No data
HAQ<br>6 months	1.56 mean	**0.21 lower**<br>1.00 lower - 0.56 higher	No data	**0.28 lower**<br>0.63 lower - 0.08 higher	**0.35 lower**<br>0.79 lower - 0.10 higher
Pain<br>6 months	58.22 mean	No data	No data	No data	**3.89 lower**<br>8.37 lower - 0.55 higher

	Placebo	Abatacept 125 mg sc Q1W	Switch in TNFi route SC->IV	Rituximab 1000 mg IV wk 0 and 2	Baricitinib 2 mg po OD
ACR50<br>6 months	84 per 1000	**179 more**<br>4 fewer - 552 more<br>⊕⊕⊕⊕	**21 more**<br>78 fewer - 518 more<br>⊕◯◯◯	**247 more**<br>75 more - 488 more<br>⊕⊕⊕⊕	**70 more**<br>22 fewer - 256 more<br>⊕⊕⊕◯
DAS28<br>8 months	6.34 mean	**1.34 lower**<br>2.43 lower - 0.22 lower<br>⊕⊕⊕⊕	**1.01 lower**<br>2.99 lower - 0.99 higher<br>⊕◯◯◯	**1.37 lower**<br>2.05 lower - 0.64 lower<br>⊕⊕⊕⊕	No data
HAQ<br>6 months	1.56 mean	**0.21 lower**<br>1.00 lower - 0.56 higher<br>⊕⊕⊕◯	No data	**0.28 lower**<br>0.63 lower - 0.08 higher<br>⊕⊕⊕◯	**0.35 lower**<br>0.79 lower - 0.10 higher<br>⊕⊕⊕◯
Pain<br>6 months	58.22 mean	No data	No data	No data	**3.89 lower**<br>8.37 lower - 0.55 higher<br>⊕⊕⊕◯

Color guide

High or Moderate Certainty

Among the most effective	Inferior to the most effective, but superior to placebo	No more effective than placebo
Among the most harmful	Less harmful than some alternatives, but more harmful than placebo	No more harmful than placebo

Low or Very Low Certainty

May be among the most effective (or harmful)	May be inferior to the most effective, but superior to placebo (or less harmful than some alternatives, but more harmful than placebo)	May be no more effective (or harmful) than placebo

Color guide

High or Moderate Certainty	Low or Very Low Certainty
Superior to placebo, but not superior to any other effective intervention	May be superior or inferior to placebo, but no more superior or inferior than any other treatments
No more effective or harmful than placebo	May be no more effective (or harmful) than placebo
More harmful than placebo, but no more harmful than any other harmful intervention	May be superior or inferior to placebo, but no more superior or inferior than any other treatments

3rd layer

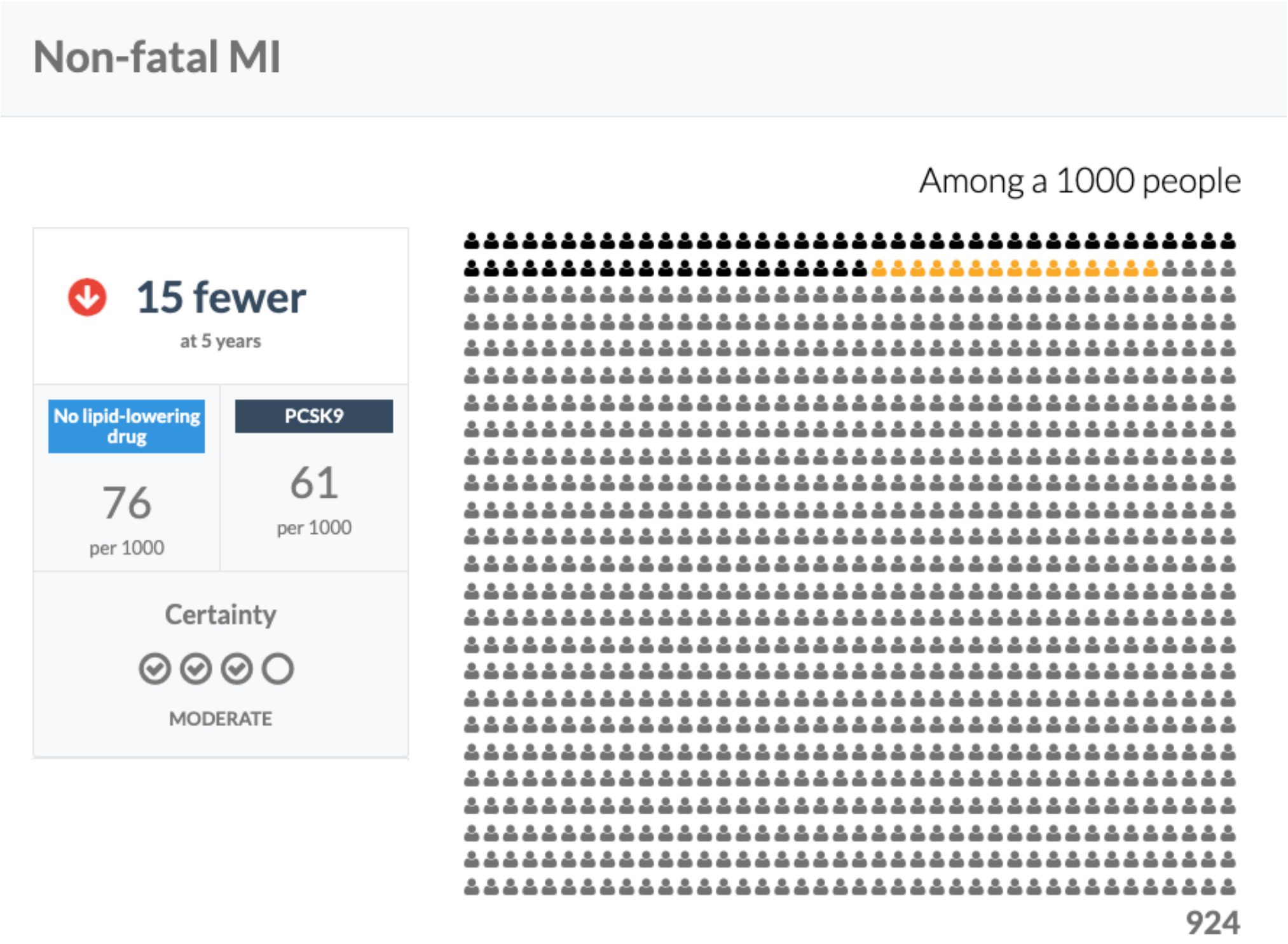

Non-fatal MI
Among a 1000 people
15 fewer
at 5 years
No lipid-lowering drug
76
per 1000
PCSK9
61
per 1000
Certainty
MODERATE
924

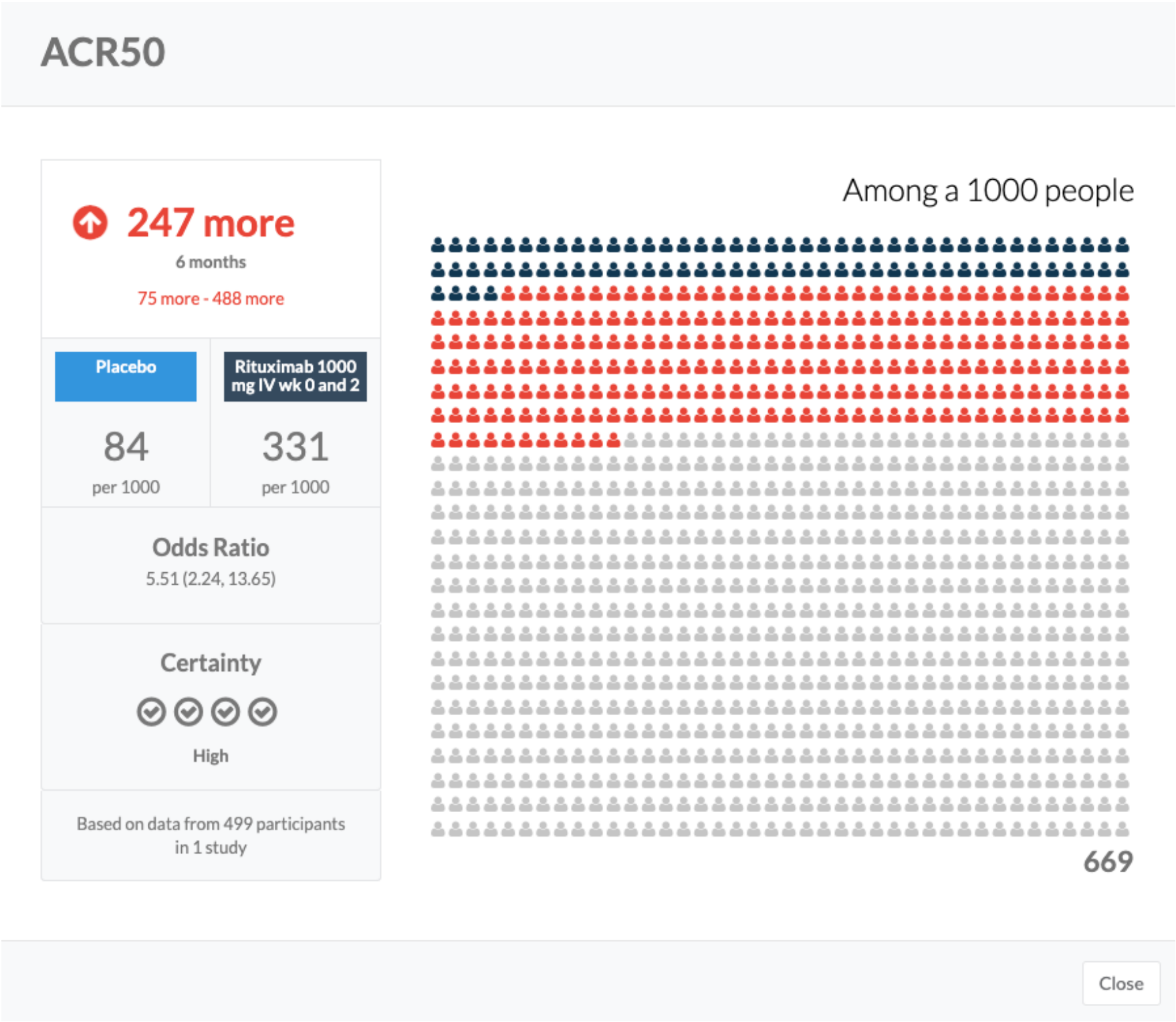

ACR50
247 more
6 months
75 more - 488 more
Placebo
84
per 1000
Rituximab 1000 mg IV wk 0 and 2
331
per 1000
Odds Ratio
5.51 (2.24, 13.65)
Certainty
High
Based on data from 499 participants in 1 study
Among a 1000 people
669
Close

Practical issues

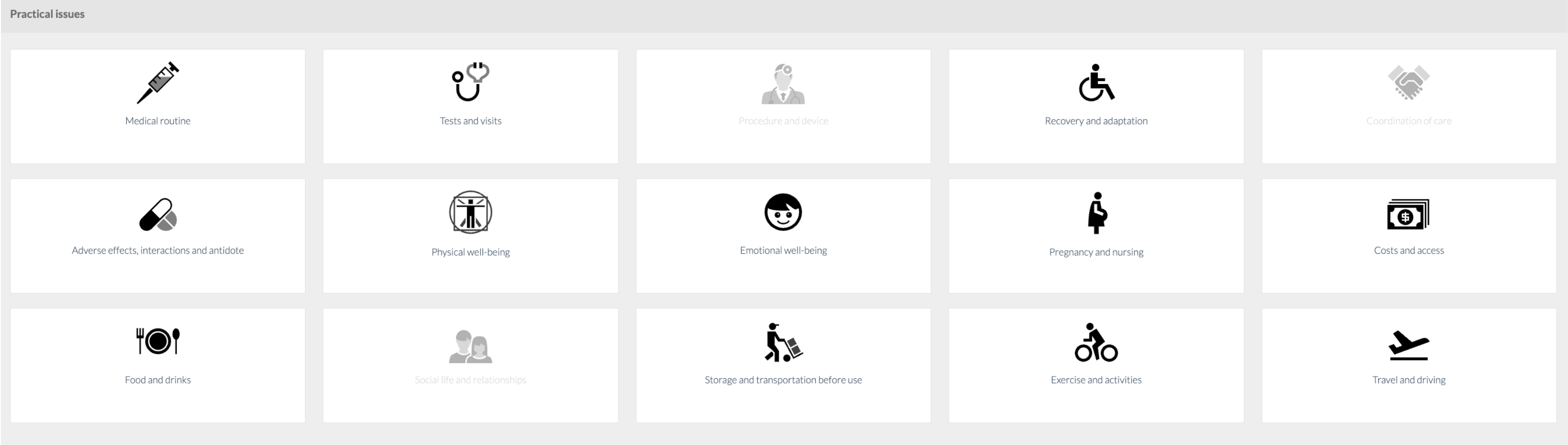

**Dosing**	N/A	No change.	Subcutaneous injections weekly OR intravenous infusions every 4 weeks.	Pills once daily.	Intravenous, 2 infusions every 8 weeks.	Intravenous infusions every 8 weeks.	Pills once daily.
**FDA black box warnings**	N/A	No new risks. Warnings with anti-TNF therapy include: serious infections and malignancy.	No black box warnings.	JAK inhibitors: serious infections, mortality, malignancies, major adverse cardiovascular events and thrombosis.	Infusion-related reactions, mucocutaneous reactions, hepatitis B virus reactivation and progressive multifocal leukoencephalopathy.	No new risks. Warnings with anti-TNF therapy include: serious infections and malignancy	JAK inhibitors: serious infections, mortality, malignancies, major adverse cardiovascular events and thrombosis.
