# Supplementary material for: MATCH‐IT: A decision support tool for multiple comparisons presenting data from network meta‐analysis to facilitate guideline development
Source: Clin Public Health Guidel. 2024 Sep 17;1(4):e70003. doi: 10.1002/gin2.70003 (PMC13563384; doi:10.1002/gin2.70003)
Supplement: Supplementary file 2 [file GIN2-1-e70003-s002.pdf]

Code	Subtheme	Theme	Category
Addition of colour code facilitate a clearer communication of the effects of interventions	Categorization of results with colour code facilitate clear communication of effects, makes it easier to get an overview of the evidence, and its a clear preference that colour code indicate the direction of effects for both levels of certainty	Reporting of effects in absolute measures with certainty are appreciated, categorization with colour code enhance clarity	Strengths
Appreciate that colour codes indicate direction of effects for both high/moderate and low/very low certainty			
Appreciate the colour code only differentiate certainty in two categories. Risk of the colour code to be too complex with four certainty categories			
Appreciate the colour coding, it creates colour patterns in the SoF table that was useful			
Colour code facilitated interpretation of results in the SoF table during the panel meeting			
Colour code helps communicate level of certainty more clearly			
Colour guide needs to be easily visible			
Prefer categorization of interventions with colour code vs no categorization			
The ability to group interventions during the discussion (using drag and drop) was helpful			
The complexity of the network in terms of categorization may influence how well/easy the colour code is to understand.			
The SoF structure in combination with colour coding provide a good overview with colour codes facilitated clear communication of effects			
Appreciate having certainty reported as text in addition to colour code	Reporting of results in absolute measures with CrI/CIs and dedicated certainty text are appreciated. Additional data elements in 3rd layer is well-received with pictogram being highly appriciated by patient representatives		
Appreciate that effects are reported in absolute measures			
Appreciate the additional data elements added to 3rd layer			
Appreciate the additional data elements added to 3rd layer, Pictogram perceived as a useful visualization of effects, but to some extent depent on context and individual preferences			
Appreciate the inclusion of plain language summaries			
Appreciate the updated pictogram window with additional data elements			
Appreciate the visualization of effects in pictograms			
For individuals with methodological competence, the tool is easy interpretable			
Pictogram intuitive to understand			
Pictogram perceived as a useful visualization of effects, but to some extent depent on context and individual preferences			
Preference for certainty ratings reported as text rather than symbols			
Some might find it useful with dedicated certainty reporting in addition to colour code			
Useful to have CrIs/CIs displayed to show the imprecision			
Valuable in a guideline process to have information about primary studies included in the tool			
Appreciate the ability to view results across multiple outcomes	The SoF table provide a good overview without being cluttered with too much details, it´s well balanced focusing on key information elements and may facilitate discussions. The opportunity to drill down for more details is appreciated and PI information structure is perceived useful	The table structure provide a good overview of the evidence, the layer structure with ability to drill down for more information is appreciated, and practical issues are useful to include	
Appreciate the PI grid structure, ability to easily find and target specific information needs			
Layering of the tool is fine			
No superfluous information in the tool			
Positive reaction to the PI-card, preference for this solution vs grid			
Positive to the idea of drill down in the evidence, including GRADE SoF for pairwise comparisons may be useful			
Positive to the idea of drill down in the evidence, layer by layer			
Reporting of absolute effects are clear and the combination of displaying results across multiple outcomes with dedicated certainty reporting makes it easy to get an overview			
The SoF structure provide a good overview of the evidence			
The SoF structure provide a good overview of the evidence, may facilitate discussions during guideline meetings			
The SoF structure, with few details displayed provide a good overview of the evidence that is easy to interpret			
The SoF table is well balanced, providing overview without overload of details			
The SoF table structure with reporting of absolute effects provide a good overview that is easy to interpret			
The tool is well balanced, providing overview without overload of details			
The tool provide a good overview of the evidence			
The tool provide a high level summary of the evidence			
Immediate positive reaction to PI. Useful.	Useful to have PI information included, highlight relevant and important aspects to consider both in a guideline and clinical context		
PI information highlight important aspects to consider in a guideline process			
PI information highlight important aspects to consider in a guideline process, positive response to PI-card			
PI information highlight relevant and important aspects to consider for a decision			
PI information is relevant to include in a guideline process, may facilitate discussions on recommendations			
PI information is useful and it´s communicated in a clear way			
PI information may be relevant to consider in contexts where interventions are quite similar in terms of effects for benefits and harms			
PI information is key to consider for clinical decision-making			
Useful to have PI information included			
Useful to have PI information included, categories of interest may vary			
Useful to have PI information included, highlight relevant and important aspects to consider in a guideline context			
Useful to have PI information included, highlight relevant and important aspects to consider in a guideline that maybe not all know			
Appreciate fonts and colours used	Appreciate the visual design of the tool	Quite easy to use, navigate and understand.	
General positive reaction to the tool, looks very good			
Appreciate the ability to navigate with the "show all/hide all" button	Navigation in the tool is intuitive, the inclusion of "short-cuts" such as "Show all" outcomes button is both used and perceived useful		
Appreciate the fluent navigation of the tool			
Navigation of the tool feels intuitive			
Use "show all/hide all" button as intended			
Appreciate the new tutorial ability to provide answer			
General positive reaction to the tool, user friendly			
PI information is easy understandable from a layperson perspective			
Probably useful to keep a consist user-interface across MAGIC products			
Quite easy to learn to use and understand			
The tool communicate science in a language clinicians can understand			
The tool is easy to understand			
The tool is perceived as easy to understand			
Watching tutorial enables use of interactive features			
Browsing practical issues	Users able to fluently build SoF, correctly interpret information in SoF and pictogram, fluently browse PI information.		
Browsing practical issues information			
Build SoF			
Build SoF and interpret information			
Coping with concepts of colour coding and understand colour guide			
Coping with concepts of drag and drop in SoF			
Coping with concepts of PI			
Decide to view tutorial upfront			
Draw conclusions based on information in SoF			
Interpret and understand tutorial			
Interpret information in 1st layer and start selecting outcome			
Interpret information in 1st layer and start selecting outcomes, building SoF and interpret information			
Interpret information in 1st layer, coping with the concept of the tool			
Interpret information in 1st layer, start selecting outcome and interpret information			
Interpret information in 3rd layer/pictogram			
Interpret information in SoF			
Interpret information in SoF, access 3rd layer spontaneously			
Interpret risk selection site and act accordingly			
Manouver fluently between layers in the tool			
Open FAQ and interpret information			
Use the tool with all functions fluently after observed use of the tool in a panel meeting prior to user-testing			
Appreciate that the SoF table can be built step-by-step, good approach in guideline meetings	Interactive features are useful. Ability to build SoF, change comparator and in other ways adjust the SoF are positively appraised and relevant in a guideline setting. Users are able to easily use the SoF functionality	Interactivity, dynamically building and adjusting the SoF table are valuable features in guideline meetings	
Change comparator and continue to build SoF			
Changing comparator has value, allow for better understanding			
Changing comparator has value, allow for better understanding, but not intuitive to discover			
Interactive features provides value, making the tool more flexible/adjustable			
Interactive features provides value, making the tool more flexible/adjustable, but not intuitive to discover			
Interactive features provides value, making the tool more flexible/adjustable. Able to use the function intuitively after demo in panel meeting			
Positive reaction to the ability of moving interventions			
Understand how to change comparator			
Use drag and drop functionality of SoF			
Valuable to be able to group interventions in the SoF table			
Assessing the evidence may be complicated and this tool may help facilitate the learning process	The tool provided value in a guideline meeting or is percieved to be potentially useful in guideline meetings. It communicate complex evidence clearly in a manageble format, facilitating understand for laypersons and may help improve discussions		
General positive reaction to the tool			
The tool help laypersons in identify what´s key information, making science more accessible			
The tool may be useful in panel meetings, assessing the evidence			
The tool was in general very good			
The tool was useful in assessing the evidence in the panel meeting. It facilitated communication of complex evidence in a way that the panel clearly understood, allowing for display multiple comparisons across several outcomes in one overview			
The tool was useful in assessing the evidence in the panel meeting. Panel member used it on the side			
The tool was useful in assessing the evidence in the panel meeting. Seemingly it communicated complex evidence in a way that the panel clearly understood			
The tool would be useful to have in panel meetings, assessing the evidence			
Tool is most probably useful to apply in guideline setting			
PI may facilitate shared decision-making	The tool is perceived potentially useful at the point of care, especially in communication with patients (in SDM)	The tools is perceived as potentially valuable also at the point of care, especially for shared decision making	
Pictogram perceived as a useful visualization of effects in patient communication			
The tool may facilitate shared decision-making			
The tool may facilitate shared decision-making and can be used in lecturing as well			
The tool may inform decision-making at the point of care			
Would apreciate use of MATCH-IT for other treatment indications as well			

Category	Theme	Subtheme	Code
Problems	Suboptimal colours/contrast, ambiguous or suboptimal visibility of graphical elements	Colours perceived to be too neutral, contrast for some graphical elements are too little, non-corresponding colours between linked graphical elements or negative reactions to use of symbols	Choice of colours perceived too neutral, boring or depressing
			Colour guide that includes the use of white blends in with the background and makes it difficult to interpret
			Contrast in comparator column is suboptimal
			Negative reaction to the use of wheelchair icon for the "recovery and adaptation" category in PI
			Non-corresponding colours between pictogram and effect estimates
		Suboptimal visibility of graphical elements	Colour guide is may be hidden when it is placed below the screen
			Not sufficiently visually clear which baseline risk that is currently selected
	Suboptimal precision of information in practical issues and other language issues.<br>The value of pictograms in guidelines are less clear	Concerns with the general concepts of NMA	Concerns with the general concept of NMA that all interventions can interchangeably be compared
		In context of guideline development the value of interactive features in SoF and visualization of effects in pictograms are less clear	The user does not see the immediate value of drag and drop interactivity in SoF
			Visualization of effects with pictograms may well be useful in other contexts, but not very useful in a guideline development process
		Precision of language or content in PI is not sufficient, use of abbreviations or normative terms in reporting of effects or certainty of the evidence are a source for confusion	Miss information about treatment indications for the different interventions
			Not clear what N/A in the certainty column imply
			Not intuitive to understand the content within a PI category based on the icon and label
			PI information is not consistent, sometimes medication specific, other times disease specific
			Precision of PI information is not sufficient according to the user´ information need
			The use of "no important difference" for no difference is confusing and to some extent normative
	Difficulties with discovery of some interactive features and other navigation issues	Difficulties with navigation between layers, discovery and/or use of interactive features in the SoF-table or the tool more broadly	Confusing with the mix of drag and click. Drag out, click in.
			Difficulties to navigate back to risk selection
			Difficulties with adding back interventions to the SoF table
			Not intuitive how to, rearrange or remove interventions/outcomes from the SoF table
			Not intuitive that a 3rd layer can be accessed, not sufficiently clear that the effect estimate cells are clickable
			Not intuitive that elements are clickable
			Not intuitive that you can view multiple outcomes at once
	Tutorial perceived as suboptimal, miss guidance for use of the tool.	Tutorial module lack audio support and is running too fast	Tutorial is less informative without audio support
			Tutorial video is running too fast
		Miss information on purpose of and/or instructions for use of the tool	Miss information that provide more context for what the tool is and for who it is ment to serve
			Miss some more explanation on the visualization of effects in 3rd layer
			Miss some more guidance/instructions in use of the tool
		Unfamiliar with the concept of certainty of the evidence or visualization of effects in pictograms	Unfamiliar with the concept of certainty of the evidence and what it include
			User not immediately convinced about the accuracy of the pictogram (is it really 1000 people displayed?)
	Programming issues, bugs and typos	Manual data important is a barrier for use. It takes too much time to import all data variables, which again are vital for having a fully functional tool to display	If CIs and additional details in 3rd layer is to be used it needs to be automatically updated when changing the comparator
			Manual input of data is very time consuming and a barrier for use
		Program bugs or typos	Clickable element does not respond or the element is looking clickable by mistake
			Colour code is not updated as it´s supposed to
			Interventions removed from SoF lies above and cover cells in the remaining part of the SoF
			Lack of automatic scaling of visual elements when zoom in/out in browser
			Typo in PI
	Miss the display of effects´ imprecision and access to more details usually found in pairwise SoFs. Evaluation of certainty and method for categorization are not communicated in sufficient detail.	Miss definition of terms, more detailed information about primary studies, transparency in reporting of certainty, source for PI information, imprecision measures for estimates of effects and reporting of AEs in SoF.	Adverse effects should be reported in SoF with effect estimates, not only as text in PI
			Miss a reference to the information source/more information on the origin of the evidence to provide more context
			Miss clear definition of diagnostic marker
			Miss clear definition of risk groups
			Miss information on details from primary studies included
			Miss information on subcomponents of the effect estimates, what is direct and network effects?
			Miss the reporting of CrIs/CIs, display of imprecision
			Miss transparency in how certainty of the evidence is assessed
			Miss transparency with reference to the source(s) of PI information
		Rationale for categorization of interventions are not clear or perceived as too complicted to grasp, colour coding are either not adapted to the data set or it may conflict a neutral interpretation	Colour code is not adapted to the data displayed
			Colour code may draw attention and conflict neutral interpretation of the evidence
			Rationale behind the colour code is not clear to user
			The rationale behind categorization may be too complicated to understand without any form of preparation
		Reporting of effects do not sufficiently convey imprecision or the graphical information structure is perceived suboptimal	Suboptimal graphical layout/information structure
			The tool does not sufficiently convey the imprecision of effects
Suggestions for improvement	Measures to adapt the tool to a clinical context	Update tool structure to a flow-chart structure in line with med.calcs	Update the tool to a flow-chart structure in line with med.calcs
	Measures to increase customization of the tool to better serve the different needs in a guideline meetings	Enable author/user editing/cumstomization of the tool, integrate the tool with other MAGIC modules and make the tool accessible at platforms used at the point of care	Enable user edits/customization in the tool
			Enable user pre-sets
			Integrate with other MAGIC modules for authors to start EtD processes
			Make the tool accessible to clinicians/integrate in platforms used by clinicians
		Opportunity to compare interventions across multiple risk groups, possibility to display SoFs for pairwise comparisons and have gists of PI	Highlight differences between interventions in PI
			Include a risk calculator for deciding baseline risk or more of a flow-chart structure
			Include possibility to compare outcomes across several risk groups
			Include the ability to view SoFs for pairwise comparisons
			Make PI framework more visible
			Provide gists of PI information
			Rate the importance of different PI categories for that particular data-set
	Measures to facilitate interpretation of the evidence	Include categorization of data with colour codes and filter functionality that ideally is dynamically updated with change of comparator. Include possibility to use partial or fully contextualized approach for such categorization.	Ability to dynamically update categorization when changing comparator
			Highlight drug class by colour code interventions in headline
			Include categorization with colour codes
			Include filter functionality, e.g. Drug class/category
			Include possibility to categorize results based on a partial or full contextualized model
			Suggest to have a predetermined order of outcomes
		Possibility to adjust denominator according to incidence, include reporting of CrIs/CIs, toggle function for view of data elements in SoF and report results in absolute risk instead of absolute risk difference	Colour code CrIs/CIs according to direction and crossing of tresholds
			Convert continous outcomes into dichotomous outcomes
			Do not include possibility for NNT/NNH
			Include ability to report results as NNT/NNH
			Include CrIs/CIs
			Include PI information to the data-set
			Include possibility to display results without certainty reporting
			Make denominator adjustable
			Report results in SoF in absolute risk instead of absolute risk difference
			Toggle button for display of certainty and CrIs/CIs
	Measures to improve clarity of design elements and text	Dynamically scaling of the tool, update of fontsize and colour to highlight data elements, graphical updates of colour guide to increase clarity and suggestions for update of text	Add more colours to the tool
			Asign every interventions with its own colour in SoF
			Change wording of headline in risk selection
			Colour code data elements that should stand out in 3rd layer
			Demarcate the colour guide
			Dynamic scaling of the tool according to both data amount and the device to display
			Include a label for absolute effects in 3rd layer
			Increase contrast of text elements in the comparator column.
			Increase font size
			Increase font size of heading in 1st layer
			Make plain language summary more prominent
			Update design of colour guide. Benefits to harms in one row instead of two
			Update heading of 1st layer
	Measures to improve efficient use and understanding of the tool´ purpose and functionality	Provide information on purpose of tool, instructions for use of the tool (push or pull),updates in tutorial module and updates to improve navigation	Change tutorial to step-by-step guidance
			Include audio in tutorial
			Include push/pull revelations to guide users in use of the tool
			Include route to go back in the tool
			Provide more contextual support to the colour guide
			Slow down speed of tutorial
			Subtitling of tutorial
	Measures to further increase transparency in reporting of the evidence	Include definitions and/or explanations of key terms, links/citations to source of the information and make more detailed information from the NMA available in the tool and always provide numerical data on adverse effects	Adverse effects should be reported numerically
			Include an explanation/introduction to the rationale/methodology for colour coding
			Include an explanation/introduction to the term certainty
			Include definition of outcomes
			Include information about contraindications in PI
			Include information on co-administered drugs for the patient populations
			Include links/references to primary studies
			Include more detailed information about the certainty evaluation
			Include more information about GRADE for non-experts
			Include more specific information about cost in PI
			Include possibility to access more information from the NMA and its primary studies
			Include possibility to access more information from the NMA and its primary studies with links
			Include possibility to access more information from the NMA and its primary studies, including more information on certainty evaluation and CrI/CIs
			Include reporting of relative effects
			Provide clear definition for risk groups
			Report harms in numbers in SoF
			Use standard phrases/categories according to prespecified systems in PI
