# Supplementary material for: MATCH‐IT: A decision support tool for multiple comparisons presenting data from network meta‐analysis to facilitate guideline development
Source: Clin Public Health Guidel. 2024 Sep 17;1(4):e70003. doi: 10.1002/gin2.70003 (PMC13563384; doi:10.1002/gin2.70003)
Supplement: Supplementary file 3 [file GIN2-1-e70003-s003.pdf]

Interview guide MATCH-IT for guideline developers – 2nd Group

MATCH-IT for guideline developers		
**Participants no**		
**Age and sex**		
**Location**		
**Date**		
**Interviewer/Observer**		

Checklist digital meeting

**Interviewer**	
Hyperlink to the MATCH-IT tool	☐

**Observer**	
Notebook and pen	☐

Checklist physical meeting

**Interviewer**	
A tablet or laptop with access to the MATCH-IT tool	☐
Paper prints of all layers in the MATCH-IT tool	☐

**Observer**	
Notebook and pen	☐
Video camera/audio recorder	☐

Introduction

Go through the letter of consent together with the participant.

- What is the purpose and objective of the study?
- How the user-testing is conducted.
- What happens to the data collected?
- Rights to quit or retract the recording.
- Questions?

START VIDEO/AUDIO RECORDING

Background information

A.	**Ask:** *What is your assigned role and/or which group do you represent in this guideline panel?*

B.	**Ask:** *What is your profession/occupation? Do you have academic experience beyond a Master of Science or equivalent to this?*
Profession	
Ph.D	☐ Yes ☐ No
PostDoc	☐ Yes ☐ No
Other	

C.	**Ask:** *What is your current position(s)?*

D.	**Ask:** *Do you have any clinical experience? If yes, for how many years?*
	☐ Yes<br>☐ No
Years	

E.	**Ask:** *Do you have any previous experience with guideline development? If yes, please elaborate briefly.*
	☐ Yes<br>☐ No
Details	

Repeat instructions

The interviewer will give the following instructions before the interview:
I am going to ask you a set of questions: *You are not being tested. It´s our tool we are testing. We are seeking knowledge of the users' experiences with the tool´s design, user interface and features among others. There are no right or wrong answers to our questions. We want to know if you find something easy or difficult, clear or confusing, understandable or incomprehensible.*

Think aloud! Tell me what you're thinking, what you see and what you find confusing or surprising, even the least little bit. If there's something you don't understand, just say "I don't know what this means..."

My role is to ask questions. Otherwise, I will say as little as possible since it's your opinion we're interested in.

Okay?

If not a digital meeting: START SCREEN RECORDING on the tablet/computer now

Questions

**1.**	**Ask:** *What is your overall first impression of the tool after using it during this guideline recommendation process?*

**2.**	**Ask:** *We would like to know whether such a tool is understandable to you, please tell us what you think (if necessary, follow up with questions targeting specific layers of the tool; was the 1. layer understandable to you? Etc.*

**3.**	**Ask:** *We would like to know whether such a tool is useful for you, please tell us what you think? (if necessary, follow up with questions targeting every layer of the tool; was the 1. Layer useful…? Etc)*

**4.**	**Ask:** *Is there any information lacking? (if necessary, follow up with questions targeting every layer of the tool; was there information lacking in the 1. layer? Etc)*

**5.**	**Ask:** *Is there information which you find superfluous (if necessary, follow up with questions targeting every layer of the tool; superfluous information in the 1. layer? Etc)*

**6.**	**Ask:** *Do you have suggestions for changes or improvements? (if necessary, follow up with questions targeting every layer of the tool; suggestions for changes in the 1. layer? etc)*

**7.**	**Ask:** *If you could decide; would you change the visual design, such as the font or the colours?*

**8.**	**Ask:** *If you could decide; would you organize the information layers you have seen differently? In that case: In which order do you feel they could be more helpful?*

**9.**	**Ask:** *Was there anything that particularly confused or frustrated you, or things you didn't like? (if necessary, follow up with questions targeting every layer of the tool; was there anything particularly confusing in the 1. layer? Etc)*

**10.**	**Ask:** *Was there anything that you especially liked?*

**11.**	**Ask:** *In guideline process such as the one you have been through, do you see a role for such a tool?*

**12.**	**Ask:** Throughout this BMJ RR you have used both traditional GRADE SoF-findings tables and the MATCH-IT tool. You have also used the MATCH-IT tool now. Do you have any preference for either format? If so, why?

**13.**	**Ask:** *Let's say you were in charge developing such a tool and you had no restrictions; how would you design this tool to maximise the value and usefulness for you and your colleagues in your daily practice? (If not a digital meeting: Offer the participants pen and paper, maybe also prints of the tool if asked for)*

1.	***Checklist to interviewer***
Have you during this interview covered and collected information about all relevant aspects of and features in the MATCH-IT tool?<br>☐ Tutorial<br>☐ Practical issues<br>☐ Interactivity<br>☐ Ranking/colour coding<br>☐ Certainty<br>☐ Use of "sjatteringer"<br>☐ GRADE SoF-embedded in the tool<br>☐ Third layer<br>☐ Interactivity	

14.	**Say:** *Thank you very much – that's all. But we also would like your feedback on how we might have organised this session better. Any suggestions for improving the user testing?*

Thank you for your contribution

STOP AUDIO/VIDEO RECORDING
